# Ascl1 phospho-site mutations enhance neuronal conversion of adult cortical astrocytes *in vivo*

**DOI:** 10.3389/fnins.2022.917071

**Published:** 2022-08-18

**Authors:** Hussein Ghazale, EunJee Park, Lakshmy Vasan, James Mester, Fermisk Saleh, Andrea Trevisiol, Dawn Zinyk, Vorapin Chinchalongporn, Mingzhe Liu, Taylor Fleming, Oleksandr Prokopchuk, Natalia Klenin, Deborah Kurrasch, Maryam Faiz, Bojana Stefanovic, JoAnne McLaurin, Carol Schuurmans

**Affiliations:** ^1^Sunnybrook Research Institute, Toronto, ON, Canada; ^2^Department of Biochemistry, University of Toronto, Toronto, ON, Canada; ^3^Department of Laboratory Medicine and Pathobiology, University of Toronto, Toronto, ON, Canada; ^4^Department of Medical Biophysics, University of Toronto, Toronto, ON, Canada; ^5^Department of Medical Genetics, Cumming School of Medicine, Hotchkiss Brain Institute, Alberta Children’s Hospital Research Institute, University of Calgary, Calgary, AB, Canada; ^6^Department of Surgery, University of Toronto, Toronto, ON, Canada

**Keywords:** proneural bHLH transcription factors, phospho-site mutations, neuronal reprogramming, cerebral cortex, astrocytes, induced neuron, adeno-associated virus, glial fibrillary acidic protein

## Abstract

Direct neuronal reprogramming, the process whereby a terminally differentiated cell is converted into an induced neuron without traversing a pluripotent state, has tremendous therapeutic potential for a host of neurodegenerative diseases. While there is strong evidence for astrocyte-to-neuron conversion *in vitro, in vivo* studies in the adult brain are less supportive or controversial. Here, we set out to enhance the efficacy of neuronal conversion of adult astrocytes *in vivo* by optimizing the neurogenic capacity of a driver transcription factor encoded by the proneural gene Ascl1. Specifically, we mutated six serine phospho-acceptor sites in Ascl1 to alanines (Ascl1*^SA^*^6^) to prevent phosphorylation by proline-directed serine/threonine kinases. Native Ascl1 or Ascl1*^SA^*^6^ were expressed in adult, murine cortical astrocytes under the control of a glial fibrillary acidic protein (GFAP) promoter using adeno-associated viruses (AAVs). When targeted to the cerebral cortex *in vivo*, mCherry^+^ cells transduced with AAV8-GFAP-Ascl1*^SA^*^6^-mCherry or AAV8-GFAP-Ascl1-mCherry expressed neuronal markers within 14 days post-transduction, with Ascl1*^SA^*^6^ promoting the formation of more mature dendritic arbors compared to Ascl1. However, mCherry expression disappeared by 2-months post-transduction of the AAV8-GFAP-mCherry control-vector. To circumvent reporter issues, AAV-GFAP-iCre (control) and AAV-GFAP-Ascl1 (or Ascl1*^SA^*^6^)-iCre constructs were generated and injected into the cerebral cortex of Rosa reporter mice. In all comparisons of AAV capsids (AAV5 and AAV8), GFAP promoters (long and short), and reporter mice (Rosa-zsGreen and Rosa-tdtomato), Ascl1*^SA^*^6^ transduced cells more frequently expressed early- (Dcx) and late- (NeuN) neuronal markers. Furthermore, Ascl1*^SA^*^6^ repressed the expression of astrocytic markers Sox9 and GFAP more efficiently than Ascl1. Finally, we co-transduced an AAV expressing ChR2-(H134R)-YFP, an optogenetic actuator. After channelrhodopsin photostimulation, we found that Ascl1*^SA^*^6^ co-transduced astrocytes exhibited a significantly faster decay of evoked potentials to baseline, a neuronal feature, when compared to iCre control cells. Taken together, our findings support an enhanced neuronal conversion efficiency of Ascl1*^SA^*^6^ vs. Ascl1, and position Ascl1*^SA^*^6^ as a critical transcription factor for future studies aimed at converting adult brain astrocytes to mature neurons to treat disease.

## Introduction

Neurological diseases are most often associated with the loss or dysfunction of specific neuronal populations. Once lost, neurons are not replaced, except in rare circumstances and in restricted brain niches (Grade and Gotz, 2017; Barker et al., 2018). The lack of a regenerative response, combined with a paucity of neurotherapeutics, has prompted the exploration of various neuronal replacement strategies, including exogenous cell transplants and the stimulation of endogenous neural stem cells. However, these approaches have yet to result in sufficient neuronal integration for long-term functional recovery (Adams and Morshead, 2018; Ruddy and Morshead, 2018). Moreover, introducing exogenous human cells, especially fetal stem or progenitor cells, raises ethical concerns, and may be confounded by immune rejection, tumorigenicity, and supply constraints. Identifying an *endogenous* neuronal repair strategy in which new neurons functionally integrate into existing neural circuitry would be transformative as it would provide new therapeutic strategies to treat neurodegenerative disease.

We have begun to exploit the potential of direct neuronal reprogramming for endogenous neuronal replacement (Bocchi et al., 2021; Vasan et al., 2021). This feat exploits decades of research into the roles of lineage-specifying basic-helix-loop-helix (bHLH) transcription factors (TF) in driving subtype-specific neurogenesis in the embryonic brain (Grade and Gotz, 2017; Vasan et al., 2021). The proneural bHLH TFs, including Neurog2, Ascl1 and Neurod4, and downstream bHLH genes, such as Neurod1, have emerged as critical architects of neurogenesis in the embryonic brain (Oproescu et al., 2021) and are now being exploited to drive neuronal conversion of heterologous cell types (Bocchi et al., 2021; Vasan et al., 2021). During development, proneural bHLH TFs act at the top of transcriptional cascades, turning on other TFs, such as Neurod1, which function at later developmental stages to control neuronal differentiation. However, bHLH TFs are not active in all cellular contexts and can be inhibited by environmental signals. For example, in the embryonic cortex, Neurog2 is only sufficient (by gain-of-function; Li et al., 2012) and necessary (by loss-of-function; Fode et al., 2000; Schuurmans et al., 2004; Britz et al., 2006) to specify a glutamatergic neuronal fate between embryonic day (E) 11.5 to E14.5, despite continued expression at later stages during the neurogenic period, which ends at E17. Similarly, Ascl1, which specifies a GABAergic interneuron fate in the embryonic ventral telencephalon (Casarosa et al., 1999), can only induce ectopic GABAergic genes in dorsal telencephalic progenitors at early (E12.5) and not late (E14.5) embryonic stages (Fode et al., 2000; Schuurmans et al., 2004; Britz et al., 2006).

The cell context-dependent activities of the proneural genes extend to neuronal reprogramming where there is growing consensus that the conversion of somatic cells to an induced neuron (iNeuron) fate is more efficient when the starter cell is more similar in identity (i.e., neural lineage). Thus, to efficiently convert distantly related fibroblasts to iNeurons, Ascl1 is combined with other TFs, as in the initial “BAM” combination (Brn2/Pou3f2, Ascl1, and Myt1l) (Vierbuchen et al., 2010; Wapinski et al., 2013). In this context, Ascl1 plays a crucial role as a pioneer TF, opening chromatin associated with a specific trivalent signature (H3K4me1, H3K27ac, and H3K9me3), which is then accessed by Brn2 and other neurogenic TFs (Wapinski et al., 2013). Other studies have reported that Ascl1 can convert fibroblasts to iNeurons directly, but the maturation of these iNeurons is limited (Chanda et al., 2014). Similarly, Ascl1 can trigger human pericytes to transdifferentiate into iNeurons, but only when co-expressed with Sox2, which facilitates the transiting of cells through a neural stem/progenitor cell-like stage (i.e., conversion is not direct) (Karow et al., 2012, 2018). The ability of Ascl1 to induce neural progenitor cells to differentiate into neurons is in keeping with its developmental role (Oproescu et al., 2021), and has been recapitulated using progenitor cell lines (Raposo et al., 2015) or pluripotent stem cells *in vitro*, with Ascl1 acting as a pioneer TF (Chanda et al., 2014; Yang et al., 2017; Aydin et al., 2019).

Astrocytes are common target cells for neuronal conversion as their activated state in neurodegenerative diseases and in injuries such as stroke contributes to disease pathology (Bocchi et al., 2021; Vasan et al., 2021). *Ascl1* can convert cortical astrocytes to iNeurons *in vitro*, either when misexpressed alone (Berninger et al., 2007; Heinrich et al., 2010, 2012; Kempf et al., 2021) or together with other TFs, to make for instance, dopaminergic iNeurons (Rivetti di Val Cervo et al., 2017). Spinal cord astrocytes can also be reprogrammed to iNeurons but interestingly, a distinct V2 interneuron-like identity is achieved, rather than a cortical phenotype (Kempf et al., 2021). While there are reports that Ascl1 can convert adult midbrain astrocytes to iNeurons *in vivo* (Liu et al., 2015), most studies suggest Ascl1 has low conversion efficacy in the adult cortex and hippocampus *in vivo* (Jessberger et al., 2008; Grande et al., 2013). Thus, understanding how proneural genes such as Ascl1 are regulated (i.e., inhibited), especially *in vivo*, is key for their efficient use in regenerative medicine. Several approaches have been taken to enhance the neuronal conversion efficacy of Ascl1 and Neurog2. For instance, expressing Ascl1 together with other TFs, as recently shown with a CRISPR-based approach, can enhance neuronal conversion, with resultant iNeurons having therapeutic benefits in a Parkinson’s disease model (Giehrl-Schwab et al., 2022). Similarly, *Neurog2* can be combined with other signals, such as Bcl2, to become a potent lineage converter, in part due to enhanced survival *in vivo* (Gascon et al., 2016). The knockdown of REST, a transcriptional repressor of neurogenic genes, also enhances neuronal lineage conversion (Masserdotti et al., 2015; Drouin-Ouellet et al., 2017). Finally, in another ground-breaking study, CRISPR-activation of mitochondrial genes enriched in neurons enhanced Neurog2 and Ascl1 reprogramming efficacy (Russo et al., 2021). Identifying and targeting the regulatory events that block bHLH TF activity is thus proving a fruitful strategy to improve neuronal reprogramming.

To address the challenge of lower neuronal conversion efficiency *in vivo* compared to *in vitro* (Vasan et al., 2021), we explored the importance of phosphorylation as a critical post-translational modification of Ascl1. It is now well accepted that when neurogenic bHLH TFs are expressed outside of their normal cellular context (Li et al., 2012, 2014), they are subject to phosphorylation-dependent inhibition that limits their neurogenic activity. Indeed, bHLH TF function is inhibited via phosphorylation by proline-directed serine threonine kinases (e.g., GSK3, ERK1/2, Cdks), which act in a “rheostat-like fashion;” the more serine-proline (SP) or threonine-proline (TP) sites phosphorylated, the less these TFs bind to DNA and transactivate their target genes to promote neuronal fate specification and differentiation (Ali et al., 2011; Hindley et al., 2012; Li et al., 2012, 2014). To keep bHLH TFs active, our group (Li et al., 2012, 2014) and others (Ali et al., 2011, 2020; Hindley et al., 2012; Azzarelli et al., 2015, 2022) have mutated serines (S) and threonines (T) in proline (P)-directed phospho-sites to alanines (A) (i.e., SP/TP to SA/TA mutations). These mutations prevent phosphorylation by inhibitory proline-directed kinases and increase the neurogenic potential of bHLH TFs in the embryonic mouse and frog nervous systems (Marcus et al., 1998; Ali et al., 2011, 2020; Hindley et al., 2012; Li et al., 2012, 2014; Azzarelli et al., 2015, 2022).

The goal of this study was to determine whether a mutated version of Ascl1, termed Ascl1*^SA^*^6^, is more efficient at inducing neuronal conversion of cortical astrocytes in the adult brain *in vivo.* We initiated this study using AAV and GFAP promoters (Lee et al., 2008), a combination that has since been shown to be less astrocyte-specific than initially reported due to cis-effects of TF coding sequences on the GFAP promoter (Wang et al., 2021). Nevertheless, by directly comparing Ascl1 to Ascl1*^SA^*^6^ in all of our studies, we demonstrate that compared to Ascl1, Ascl1*^SA^*^6^ has a superior capacity to induce neuronal marker expression, promote the acquisition of more elaborate dendritic arbors, and to repress astrocytic genes in the adult cerebral cortex. The enhanced capacity of Ascl1*^SA^*^6^ to induce neuronal gene expression is in keeping with embryonic studies conducted previously, and suggests that further studies of the reprogramming capacity of Ascl1*^SA^*^6^ are warranted.

## Materials and methods

### Animals and genotyping

Animal procedures were approved by the Sunnybrook Research Institute (21-757) in compliance with the Guidelines of the Canadian Council of Animal Care. In all adult animal experiments, we used male C57BL/6 wild-type mice, Rosa-ZsGreen (JAX #007906) and Rosa-tdtomato (JAX #007914) transgenic mice (Madisen et al., 2010), maintained on a C57BL/6 background, and obtained from Jackson Laboratory. For the collection of the embryonic day (E) 14.5 dorsal (dTel) and ventral (vTel) telencephalon, CD1 outbred mice were crossed and the day of the vaginal plug was considered E0.5. Mice were housed under 12-h light/12-h dark cycles with free access to food and water. PCR primers and conditions for genotyping were conducted using Jackson Laboratory protocols: Rosa-ZsGreen: wild-type forward: 5′-CTG GCT TCT GAG GAC CG-3′; wild-type reverse: 5′-AAT CTG TGG GAA GTC TTG TCC-3′; mutant forward: 5′-ACC AGA AGT GGC ACC TGA C-3′; mutant reverse: 5′-CAA ATT TTG TAA TCC AGA GGT TGA-3′. Rosa-tdtomato: mutant reverse: 5′-GGC ATT AAA GCA GCG TAT CC-3′; mutant forward: 5′-CTG TTC CTG TAC GGC ATG G-3′. PCR cycles were as follows: 94°C 2 min, 10× (94°C 20 s, 65°C 15 s *–0.5c per cycle decrease, 68°C 10 s), 28× (94°C 15 s min, 60°C 15 s, 72°C 10s), 72°C 2 min.

### Adeno-associated viruses cloning and packaging

pGFAP-mCherry-AAV (which we refer to as AAV8-GFAPlong-mCherry) and pGFAP-Mash1mCherry-AAV (which we refer to as AAV/8-GFAPlong-Ascl1-mCherry) were a gift from Leping Cheng (Liu et al., 2015) and include a 2.2 kb GFAP promoter (Zhuo et al., 1997; Liu et al., 2015). We replaced Ascl1 with Ascl1*^SA^*^6^ in AAV2/8-GFAPlong-Ascl1-mCherry. In Ascl1*^SA^*^6^, serines in all six SP sites was mutated to alanines (as in Li et al., 2014). pAAV-GFAPshort-iCre was subcloned from pAAV-GFAP-mNeurod1-T2A-iCre, a kind gift of Dr. Maryam Faiz (Livingston et al., 2020), and includes a 681 bp (gfaABC(1)D) modified GFAP promoter (Lee et al., 2008). We outsourced to GenScript to clone Ascl1-t2a-iCre and Ascl1*^SA^*^6^-t2a-iCre into AAV5-GFAPshort, and then to replace the GFAPshort promoter with the GFAPlong promoter in the AAVs from Leping Cheng (Liu et al., 2015). After cloning, all AAVs were packaged by VectorBuilder, Inc., either with AAV capsid 8 or 5. For optogenetic experiments, AAV5-EF1a-double floxed-hChR2(H134R)-EYFP-WPRE-HGFpA (catalog # 51502-AAV5) was purchased from Addgene (20298).

### Intracranial injection of adeno-associated viruses

For intracranial injections, 16-week-old male C57BL/6 mice were anesthetized using isoflurane (2%, 1 L/min; Fresenius Kabi, CP0406V2) and injected subcutaneously with an analgesic, either buprenorphine (0.1 mg/kg; Vetergesic, 02342510) or Tramadol-HCL (20 mg/kg; Chiron, RxN704598), along with Baytril^®^ (2.5 mg/kg; Bayer, 02249243), and saline (0.5 ml, Braun, L8001). A burr hole was drilled through the skull over the cortex and a stereotaxic instrument was used to identify bregma and lambda coordinates for injection. For all AAV injections, 4.8 × 10^9^ genome copies (GC) in a 1 uL total volume were delivered into the motor cortex at 0.1 μl/min over a 10 min span using a 5l Hamilton syringe with 33-gauge needle (Hamilton, 7803-07). A stereotax was used to target the motor cortex with the following coordinates (AP: + 2.15, L/M: ± 1.7, DV: −1.7). For AAV-GFAP-mCherry vectors, C57Bl/6 animals were used, while for AAV-GFAP-iCre vectors, injections were performed in Rosa-tdtomato or Rosa-zsGreen mice. Only for the Rosa-tdtomato mice was there a change in the injection paradigm - we injected 1 × 10^12^ GC/ml in a 1 uL total volume (or 1 × 10^9^ GC total) with coordinates (AP: 2.2 LM: 0.6 DV:1.0).

### Optogenetics and electrophysiology

Adeno-associated viruses-injected mice were anesthetized using 2% isoflurane and a 4 mm craniotomy was performed (from bregma: AP 1.7mm, ML + −2.15). After removal of the dura, a silicone-based polydimethylsiloxane (PDMS) window was placed over a thin layer of 1% agarose (in PBS) covering the cortical tissue. The mice were transferred to an FVMPE-RS multiphoton microscope (Olympus) and placed under a 25x/1.05NA objective lens (Olympus) while a tungsten electrode (0.255 mm Ø, A-M System) was inserted at a 30° angle, through the PDMS window, reaching a depth of 100 μm into the cortex. An Insight Ti:Sapphire laser (SpectraPhysics) tuned to 900 nm was used to excite the ZsGreen fluorescence, whose emission was then collected by a PMT aligned with a band-pass filter (485–540 nm). A second channel (575–630 nm) was also recorded simultaneously to better visualize the position of the Tungsten electrode’s tip into the tissue. For simultaneous focused photostimulation (PS) of ChR2, a raster scanned visible wave-length laser (458 nm) with a separate galvanometer was used. The PS was presented over a circular area of 250 μm in diameter where Zs-green-positive cells were present. The PS was repeated ten times over the same area at 10 s intervals (PS off). The PS was delivered over the circular area at 4 Hz with a power of 4 mW/mm^2^, and lasted for a total of 3 s. The low-impedance tungsten electrode was used to acquire voltage changes in the LFP band (1−300 Hz), recorded in current clamp mode by the Axon multiclamp 700B amplifier (Molecular devices). The analog signal was amplified 40 times (40 mV/mV) and digitized by the data acquisition system Digidata 1440A (Molecular devices). A two-phase decay model was used to describe the repolarization phase of the LFP signal, and the slower component was reported as the decay constant.

### Tissue processing and sectioning

Mice were anesthetized with ketamine (75 mg/kg, Narketan, 0237499) and xylazine (10 mg/kg, Rompun, 02169592) prior to perfusion. Intracardial perfusion was performed with approximately 20x blood volume using a peristaltic pump at a flow rate of 10 ml/min with ice-cold saline (0.9% NaCl, Braun, L8001), followed by 4% paraformaldehyde (PFA, Electron Microscopy Sciences, 19208) in phosphate buffer saline (PBS, Wisent, 311-011-CL) for 5 mins. Brains were collected and post-fixed overnight in 4% PFA in PBS, cryoprotected at 4°C in 20% sucrose (Sigma, 84097)/1X PBS overnight. Coronal brain sections were cut at 10−30 μm on a Leica CM3050 cryostat (Leica Microsystems Canada Inc., Richmond Hill, ON, Canada) and collected on Fisherbrand™ Superfrost ™ Plus Microscope Slides (Thermo Fisher Scientific, 12-550-15).

### Immunostaining

Slides were washed in 0.3% Triton X-100 in PBS, then blocked for 1 h at room temperature in 10% horse serum (HS, Wisent, 065-150) and 0.1% Triton X-100 (Sigma, T8787) in PBS (PBST). Primary antibodies were diluted in blocking solution as follows: mouse anti-Ascl1 (1:100, BD Bioscience #556604), rat anti-BrdU (1:250, Abcam #ab6326), rabbit anti-Dcx (1:500, Abcam #ab18723), goat anti-GFAP (1:500, Novus #100-53809), guinea pig-anti MAP2 (1:1000, Synaptic Systems #188 004), mouse anti-NeuN (1:500, Millipore Sigma #MAB377), and rabbit anti-Sox9 (1:500, Millipore #AB5535). Slides were washed three times for 10 min each in 0.1% Triton-X-100 in PBS, and incubated with 1:500 dilutions of species-specific secondary antibodies all from Invitrogen Molecular Probes™ for 1 h at room-temperature. Secondary antibodies conjugated to Alexa568 included goat-anti-rat (A11077) and donkey-anti-rabbit (A10042), to Alexa488 included donkey-anti-rabbit (A21206), goat-anti-mouse (A11029) or donkey-anti-goat (A11055), and to Alexa647 included goat-anti-guinea pig (A11073). Slides were washed three times in PBS and counterstained with 4′,6-diamidino-2-phenylindole (DAPI, Invitrogen, D1306). Finally, the slides were washed three times in PBS and mounted in Aqua-polymount (Polysciences Inc.,18606-20).

### RNA *in situ* hybridization

We performed colorimetric RNA-*in situ* hybridization (ISH) using a digoxygenin-labeled *Ascl1* riboprobe as previously described (Touahri et al., 2015). We performed fluorescent RNA-ISH using an RNAscope^®^ Multiplex Fluorescent Detection Kit v2 (ACD #323110) and followed the manufacturer’s instructions. Briefly, brain sections were post-fixed (4% PFA/1XPBS) for 15 min at 4°C, and then, at room-temperature, dehydrated in 50%, 70%, and 100% ethanol (Commercial Alcohols, P016EAAN) for 5 min each, and incubated in H_2_O_2_ solution for 10 min. Sections were then incubated in 1x target retrieval solution for 5 min at 95°C, washed in dH_2_O, and then incubated in Protease Plus (ACD, 322331) for 15 min at 40°C before washing in washing buffer. We used a labeled RNA probe for Ascl1 (Mm-*Ascl1* #313291) and used the negative and positive control probes provided. Sections were incubated with the probes for 2 h at 40°C. Amplification and staining steps were completed following the manufacturer’s instructions, using an Opal™ 570 (1:1500, Akoya #FP1488001KT) fluorophore.

### Western blotting

C57/Bl6 motor cortices were transduced with AAV8-GFAPlong-mCherry, AAV8-GFAPlong-Ascl1-mCherry or AAV8-GFAPlong-Ascl1*^SA^*^6^-mCherry viruses using the coordinates described above. After 7 dpi, left and right brain hemispheres were harvested, mCherry^+^ motor cortices were microdissected, and tissue was lysed in NP-40 lysis buffer (0.05 M Tris pH 7.5, 0.15 M NaCl, 1% NP-40, 1 mM EDTA, 50 mM NaF, 0.2 mM Na_3_VO_4_, 2 mM PMSF, 0.05 mM MG132, #M7449, Sigma), 1X complete protease inhibitor tablet (#04 693 116 001, Roche) for 30 min on ice. E14.5 CD1 telencephalons were collected and dissected into dorsal (dTel) and ventral (vTel) domains and similarly lysed in the same buffer. Brain lysates were centrifuged at 13,000g for 15 min and cleared supernatants were collected. Protein concentrations were quantified using a Bradford assay (#500-0006, Biorad) and a BSA protein standard. 10 μg of total protein was run on 10% SDS-PAGE gels at 70 V during stacking and 120 V while resolving. Separated proteins were transferred to PVDF membranes (#1620177, Biorad) in transfer buffer (25 mM Tris, 192 mM glycine, 20% methanol, pH 8.3) at 40 V overnight at 4°C. Membranes were blocked in TBST (10 mM Tris, 100 mM NaCl, pH 7.4, 0.1% Tween-20) with 5% (W/V) powdered milk for 1 h at room temperature and then incubated with primary antibodies diluted in the same blocking solution overnight at 4°C. Membranes were washed 3 × 10 min in TBST, and then incubated for 1 h at room temperature with 1/10,000 dilutions of horseradish peroxidase (HRP)-coupled secondary antibodies (Anti Rabbit IgG #7074S, Cell Signaling Technology, Anti Mouse IgG #Pierce 31430, Thermo Fisher Scientific) Membranes were washed 3 × 10 mins at room temperature and then processed with ECL Plus Western Blotting Reagent (#29018904, GE Healthcare) before developing with X-ray film (#1141J52, LabForce) and Biorad Chemidoc MP imaging system using Image Lab software. Primary antibodies included: 1/1000 rabbit mAb (#4695S) anti-p44/42 MAPK (Erk1/2) (137F5) (Cell Signaling Technology), 1/1000 rabbit mAb (#4370S) anti-phospho-p44/42 MAPK (Erk1/2) (Thr202/Tyr204) (D13.14.4E) XP^®^ (Cell Signaling Technology), 1/1000 rabbit mAb (#12456S) anti-GSK-3β (C5C5Z) XP^®^ (Cell Signaling Technology), 1/1000 rabbit pAb (#PA5-82086) anti-CDK1 (Thermo Fisher Scientific), 1/1000 rabbit pAb anti-β-actin (#ab8227, Abcam), 1/1000 mouse anti-ASCL1 (#556604, BD Biosciences), 1/1000 rabbit pAb (#ab74065, Abcam) anti-ASCL1and Rabbit mAb anti-phospho-Ascl1 (Li et al., 2014). Densitometry was assessed using Image J, and phospho-Ascl1 expression levels were normalized relative to Ascl1 and to β-actin.

### Phos-tag™ western blots to detect phosphorylated Ascl1

Protein lysates collected for Western blotting were de-phosphorylated by incubating with 400 units of Lambda Protein Phosphatase, with 1X NEBuffer for Protein MetalloPhosphatases (PMP) and 1 mM Mncl2 (NEB, cat# P0753S) at 30°C for 30 min. 10 μg of untreated and phosphatase-treated protein was then run at 50 mA constant current at 4°C on 10% acrylamide gels containing 20 μM Phos-tag ™ reagent (#AAL-107, FUJIFILM Wako Chemicals, United States Corporation) and 40 μM MnCl_2_ (Woods et al., 2022). Gels were washed 3 × 10 min in transfer buffer containing 10 mM EDTA followed by a final 10 min wash in transfer buffer without EDTA before transfer to PVDF membranes and Western blotting with 1/1000 rabbit pAb (#ab74065) anti-ASCL1.

### Imaging, quantification, and statistics

All images were taken using a Leica DMi8 Inverted Microscope (Leica Microsystems CMS, 11889113) with the following exceptions. Images in Figures 3D, 4B were acquired using a Zeiss Z1 Observer/Yokogawa spinning disk (Carl Zeiss) microscope. Tiled images encompassing the entire motor cortex were acquired using 30 μm z-stacks with a 1 μm step-size with a 20X objective. In Figures 3B,C, whole section images were scanned with the Zeiss AxioScan Z1 unit (Carl Zeiss Canada) using a Plan-Apochromat 10X objective and acquired with a Hamamatsu CCD camera. Figures were created with Adobe Photoshop and schematics were created with a license to BioRender.com. Statistical analyses were conducted using GraphPad Prism 9.2.0 Software. Mean values and error bars representing the standard error of the mean (s.e.m.) are plotted. Quantification of immunostained cells was performed on three brains per condition and a minimum of three sections per brain. Comparisons were made using a One-Way ANOVA and Tukey multiple comparisons. Significance was defined as p-values less than 0.05 and denoted as follows: ns, not significant, <0.05*, <0.01^**^, <0.001^***^.

## Results

### Ascl1 is phosphorylated by proline-directed serine/threonine kinases in the adult cortex *in vivo*

**FIGURE 1:**
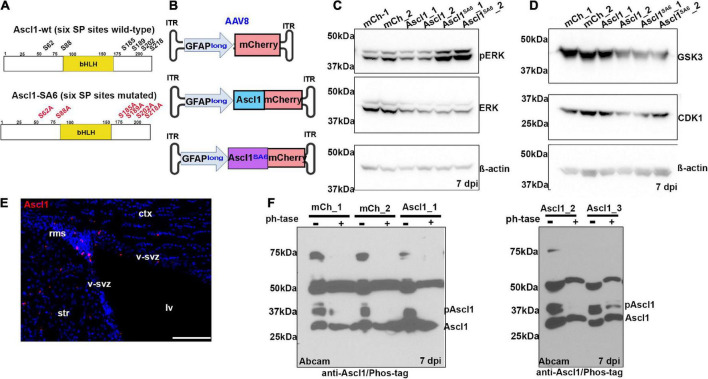
Ascl1 is phosphorylated by proline-directed serine-threonine kinases in the adult brain *in vivo.* **(A)** Schematic illustration of the sequence of wild-type (wt) Ascl1 with SP sites designated, and Ascl1*^SA^*^6^, with SA mutations indicated. **(B)** Schematic illustration of AAV8-GFAPlong-mCherry vectors. **(C,D)** Western blot analysis of lysates from right (sample 1) and left (sample 2) cortical hemispheres transduced with AAV8-GFAPlong-mCherry (mCh), AAV8-GFAPlong-Ascl1-mCherry (Ascl1) or AAV8-GFAPlong-Ascl1*^SA^*^6^-mCherry (Ascl1*^SA^*^6^), analyzed for the expression of pERK, ERK, and β-actin **(C)**, or GSK3, CDK1, and β-actin **(D)**. **(E)** Ascl1 expression in the ventricular-subventricular zone (V-SVZ) in a coronal section of the adult cortex. **(F)** Phos-tag western blot of cortical lysates from left (sample 1); left (sample 2); and right (sample 3) cortical hemispheres transduced with mCherry or Ascl1-mCherry in two independent experiments shown in separate gels either treated or not with Lambda protein phosphatase (ph’tase) and blotted with anti-Ascl1 (Abcam). Scale bars in panel **E** = 100 μm. ct, cortex; lv, lateral ventricle; rms, rostral migratory stream; str, striatum; v-svz, ventricular-subventricular zone. Significance was defined as *p*-values less than 0.05 and denoted as follows: ns, not significant, <0.05 *, <0.01 **, <0.001 ***.

Phosphorylation of Ascl1 on six SP sites (S62, S88, S185, S189, S202, and S218) by proline-directed serine-threonine kinases (e.g., ERK, GSK3, and CDK1; Figure 1A) has so far only been demonstrated in transfected HEK293 (Li et al., 2014), neuroblastoma (Woods et al., 2022), and glioblastoma (Azzarelli et al., 2022) cells *in vitro*. To determine whether Ascl1 is phosphorylated when expressed in adult cortices *in vivo*, we transduced both motor cortex hemispheres of adult C57Bl/6 mice using a stereotax to guide viral delivery (AP: + 2.15, L/M: ± 1.7, DV: -1.7). We delivered a total of 4.8^10^9^ genome copies (GC) of adeno-associated virus (AAV) 8 carrying a 2.2 kb human GFAP promoter (Zhuo et al., 1997; Lee et al., 2008) (hereafter, GFAPlong, as in Lee et al., 2008; Liu et al., 2015) to drive the expression of mCherry (control), Ascl1-mCherry or Ascl1*^SA^*^6^-mCherry fusion proteins (Figure 1B). Notably, this viral delivery system was previously used to express Ascl1 in adult midbrain astrocytes, leading to their successful conversion to iNeurons *in vivo* (Liu et al., 2015). After 7 days post-infection (dpi), left and right motor cortices were independently microdissected and analyzed for the expression of kinases that might phosphorylate Ascl1. ERK and its active pERK form (Figure 1C), as well as GSK3 and CDK1 (Figure 1D), were all expressed in the adult cortex and thus potentially available to phosphorylate Ascl1.

In the adult brain, Ascl1 is expressed in a limited number of cells, including active neural stem cells in the ventricular-subventricular zone (V-SVZ) and neuroblasts in the rostral migratory stream (RMS) (Jessberger et al., 2008; Urban et al., 2016), declining in these regions as animals age (Kaise et al., 2022). We confirmed that Ascl1 was indeed expressed in the V-SVZ and RMS by immunostaining coronal sections through the adult motor cortex (Figure 1E). To detect Ascl1 expression after viral transduction, and to assess its phosphorylation status, we performed western blotting using two polyclonal antibodies against total Ascl1 and a monoclonal antibody against Ascl1 phosphorylated on S185 (designated phospho-Ascl1) (Li et al., 2014). Ascl1-mCherry fusion proteins (∼53 kDa) labeled with a BD Biosciences antibody were overexpressed 1.5- and 7.3-fold over background levels at 7 days post Ascl1- and Ascl1*^SA^*^6^-transduction, respectively (Supplementary Figures S1A,B). A comparison of phospho-Ascl1/total-Ascl1 ratios revealed 2.1- and 10.3-fold decreases in relative Ascl1 phosphorylation after Ascl1 and Ascl1*^SA^*^6^ transduction vs. mCherry controls (i.e., endogenous Ascl1), respectively (Supplementary Figures S1A,B). These data support the contention that endogenous Ascl1 is phosphorylated on SP sites and suggest that overexpressed Ascl1*^SA^*^6^ is not phosphorylated, as we have previously shown in the embryonic cortex (Li et al., 2014).

As the phospho-Ascl1 antibody had previously only been validated *in vitro* (Li et al., 2014), to provide further support for Ascl1 phosphorylation in the adult brain *in vivo*, we subjected protein lysates to phosphatase treatment, and ran treated and untreated samples on a Phos-tag impregnated gel (Woods et al., 2022) (Figure 1F). Two prominent proteins between ∼28 and 37 kDa were labeled with anti-Ascl1 (Abcam) in the adult brain, running just above the predicted MW of 25 kDa for Ascl1 (Supplementary Figure S1D), and matching the MW of labeled proteins in embryonic day (E) 14.5 ventral telencephalic lysates (positive control; Supplementary Figure S1C). In mCherry and Ascl1-mCherry transduced brains collected in two independent experiments, western blotting with anti-Ascl1 (Abcam) revealed both a faster migrating unphosphorylated Ascl1 band just above the 25 kDa marker and a slower migrating phosphorylated band of ∼37 kDa that resolved upon phosphatase treatment (Figure 1F). Taken together, these data support the contention that Ascl1 is indeed phosphorylated in the adult brain *in vivo*.

### Ascl1*^SA^*^6^ has an enhanced ability to induce neuronal marker expression and a mature neuronal morphology compared to Ascl1

**FIGURE 2:**
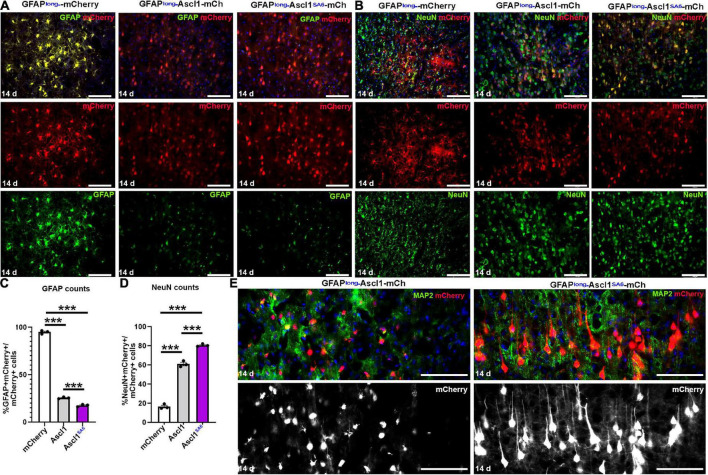
Ascl1 and Ascl1*^SA^*^6^ induce neuronal marker expression when expressed in cortical astrocytes *in vivo.* **(A,B)** mCherry co-expression with GFAP **(A)** or NeuN **(B)** 14 days post-transduction of AAV8-GFAPshort-mCherry, AAV8-GFAPshort-Ascl1-mCherry, or AAV8-GFAPshort-Ascl1*^SA^*^6^-mCherry. Blue is DAPI counterstain. **(C,D)** Quantification of the percentage of mCherry^+^ transduced cells expressing GFAP **(C)** or NeuN **(D)** 14 days post-transduction. **(E)** High magnification images of mCherry^+^ cells co-stained with MAP2 at 14 days post-transduction of AAV8-GFAPshort-Ascl1-mCherry, or AAV8-GFAPshort-Ascl1*^SA^*^6^-mCherry. Blue is DAPI counterstain. Scale bars = 100 μm. Significance was defined as *p*-values less than 0.05 and denoted as follows: ns, not significant, <0.05 *, <0.01 **, <0.001 ***.

The main goal of our study was to determine whether serine-to-alanine mutations in the six SP sites in Ascl1 would enhance neuronal conversion efficacy. Notably, our group previously demonstrated that Ascl1*^SA^*^6^ was more efficient at neuronal conversion when introduced into E12.5 cortical progenitors (Li et al., 2014), but the question remained, would this modified bHLH transcription factor more effectively convert adult cortical astrocytes to iNeurons? To determine whether Ascl1 and Ascl1*^SA^*^6^ had different abilities to induce neuronal marker expression when misexpressed in adult cortical astrocytes *in vivo*, we transduced the same set of AAV8-GFAPlong constructs into the motor cortex of C57Bl/6 mice and harvested the brains at 14 dpi (Figure 2). Packaged AAVs (4.8 × 10^9^ GC total in a 1 uL total volume) were stereotactically injected into the cortex of C57Bl/6 mice, using the same coordinates as in Figure 1 (AP: + 2.15, L/M: ± 1.7, DV: −1.7).

**FIGURE 3:**
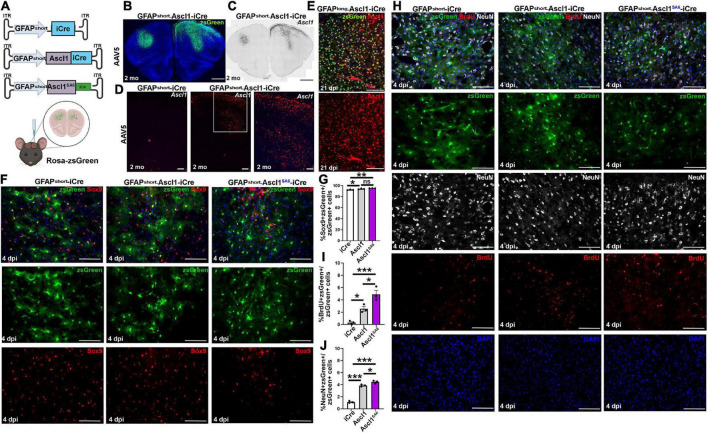
Establishing a Cre-based lineage tracing system to follow the fate of cortical astrocytes transduced *in vivo*. **(A)** Schematic illustration of AAV5-GFAPshort-iCre vectors and injection strategy into the cortex *in vivo*. **(B)** zsGreen expression in the cortex of Rosa-zsGreen mice injected with AAV5-GFAPshort-Ascl1-iCre at 2-months post-transduction. Blue is DAPI counterstain. **(C,D)** Colorimetric RNA *in situ* hybridization **(C)** and fluorescent RNAscope analysis **(D)** of Ascl1 transcript distribution in cortices transduced with AAV5-GFAPshort-iCre **(D)** or AAV5-GFAPshort-Ascl1-iCre **(C,D)** at 2-months post-transduction. The boxed area in D is magnified in the panel to the right. **(E)** Ascl1 immunostaining of motor cortex of Rosa-zsGreen animal transduced with AAV5-GFAPlong-Ascl1-iCre and harvested 21 dpi. **(F,G)** Rosa-zsGreen cortex transduced with AAV5-GFAPshort-iCre, AAV5-GFAPshort-Ascl1-t2a-iCre, and AAV5-GFAPshort-Ascl1*^SA^*^6^-t2a-iCre at 4 dpi, showing zsGreen epifluorescence and Sox9 expression **(F)**. Quantification of the percentage of zsGreen^+^ cells that co-express GFAP **(G)**. **(H–J)** Rosa-zsGreen cortex transduced with AAV5-GFAPshort-iCre, AAV5-GFAPshort-Ascl1-t2a-iCre, and AAV5-GFAPshort-Ascl1*^SA^*^6^-t2a-iCre at 4 dpi, showing zsGreen epifluorescence, NeuN (white), and Brdu (red) expression **(H)**. Blue is DAPI counterstain. Quantification of the percentage of zsGreen^+^ cells that co-express BrdU **(I)** and NeuN **(J)**. Scale bars in panels **B,C** = 200 μm, in panel **D** = 75 μm, and in panels **E,F,H** = 100 μm. Significance was defined as *p*-values less than 0.05 and denoted as follows: ns, not significant, <0.05 *, <0.01 **, <0.001 ***.

In control mCherry-transduced brains, the majority of mCherry^+^ cells had an astrocytic morphology and 95.00 ± 1.03% co-expressed GFAP (Figures 2A,C). Conversely, when Ascl1-mCherry or Ascl1*^SA^*^6^-mCherry were transduced, only 24.79 ± 0.52% and 16.31 ± 1.29% of the mCherry^+^ cells co-expressed GFAP, respectively (Figures 2A,C). To determine whether the transduced cells instead acquired neuronal marker expression, we examined the expression of NeuN, a mature neuronal marker (Figure 2B). As expected, a minor portion of control mCherry-transduced cortical cells co-expressed NeuN (14.90 ± 1.40%), while both Ascl1 (58.36 ± 1.81%) and Ascl1*^SA^*^6^ (80.75 ± 0.82%) induced 3.9- and 5.4-fold increases in the number of mCherry cells co-expressing NeuN, respectively (Figures 2B,D).

The increase in NeuN expression suggested that Ascl1*^SA^*^6^ might have an enhanced capacity to induce neuronal differentiation compared to Ascl1, as shown in the embryonic cortex (Li et al., 2014) and in glioblastoma cells (Azzarelli et al., 2022). To examine the differentiation status of these cells more closely, we examined high magnification images and co-expression with MAP2, a dendritic marker (Figure 2E). Strikingly, Ascl1*^SA^*^6^-transduced cortical astrocytes developed elaborate dendritic arbors and mature neuronal morphologies, while Ascl1-transduced cells only developed short neurite-like projections, supporting the enhanced neuronal differentiation properties of Ascl1*^SA^*^6^ (Figure 2E).

To analyze neuronal maturation at later stages, we next tried to extend the timeline of analysis to 2 months post-transduction, but at this timepoint we no longer detected mCherry expression in any of the control mCherry-transduced brains (data not shown), precluding further analyses and prompting a change in the lineage tracing system we employed.

### Adeno-associated viruses-glial fibrillary acidic protein-iCre can be used for long-term tracing of the fate of transduced cortical astrocytes *in vivo*

To circumvent the issues observed with mCherry reporter expression long-term, we turned to a Cre-based, permanent lineage tracing system. We used an AAV5-GFAPshort-iCre vector previously used in a neuronal reprogramming study to misexpress Neurod1 in cortical astrocytes (Livingston et al., 2020), replacing Neurod1 with Ascl1 or Ascl1*^SA^*^6^ to create AAV5-GFAPshort-Ascl1-t2a-iCre (abbreviated Ascl1-iCre) and AAV5-GFAPshort-Ascl1*^SA^*^6^-t2a-iCre (abbreviated Ascl1*^SA^*^6^-iCre) vectors (Figure 3A). Packaged AAVs (4.8 × 10^9^ GC total in a 1 uL total volume) were stereotactically injected into the motor cortex of Rosa-zsGreen mice, using the same coordinates as in Figure 1 (AP: + 2.15, L/M: ± 1.7, DV: −1.7). The Rosa-zsGreen and Rosa-tdtomato alleles contain floxed STOP cassettes that prevent reporter expression except in the presence of Cre, which recombines the STOP cassette out. We observed robust zsGreen expression even 2 months after viral transduction, as shown with an exemplar Ascl1-iCre transduced brain (Figure 3B).

To confirm that with this iCre-based system we could induce *Ascl1* expression in cortical astrocytes, we first examined transcript distribution in Ascl1-iCre transduced brains at 2 months dpi using RNA *in situ* hybridization with a digoxygenin-labeled *Ascl1* riboprobe (Figure 3C). Final confirmation was performed using RNAscope, which definitively showed that while *Ascl1* transcripts were not detected in the parenchyma of the adult cortex transduced with iCre control vectors, robust *Ascl1* expression was detected in the Ascl1-iCre transduced brains 2 months post-transduction (Figure 3D). Finally, we confirmed that *Ascl1* transcripts were translated into protein by immunostaining Rosa-zsGreen brains transduced with an AAV5-GFAP-Ascl1-iCre vector, revealing nuclear Ascl1 expression in the zsGreen transduced cells at 21 dpi (Figure 3E).

Next, to test the specificity of our reporter system for astrocytic labeling, we performed short-term lineage tracing at 4 dpi. AAV5-GFAPshort vectors driving the expression of iCre, Ascl1-iCre and Ascl1*^SA^*^6^-iCre were transduced into Rosa-zsGreen motor cortices and at 4 dpi, co-expression of zsGreen with Sox9, an astrocytic marker, was examined (Figure 3F). In the iCre control transduced brains, zsGreen^+^ cells had an astrocytic morphology, and the majority co-expressed Sox9 (92.6 ± 0.4%) (Figures 3F,G). Similarly, even though the astrocytic morphologies of zsGreen^+^ cells transduced with Ascl1 and Ascl1*^SA^*^6^ vectors were less pronounced, at 4 dpi, the majority of these cells expressed the astrocytic marker Sox9 (94.3 ± 0.5% and 95.5 ± 0.1%, respectively; Figures 3F,G). Notably, both Ascl1 and Ascl1*^SA^*^6^ induced small but significant 1.02- and 1.03-fold increases in Sox9 expression compared to control iCre transduction, consistent with Sox9 being an Ascl1 target gene in oligodendrocyte lineage development (Li et al., 2014). However, overall, we can conclude that for all three vectors, the majority of transduced cells are astrocytes, validating the specificity of our delivery system, at least at these early stages.

One of the questions in the field is whether astrocytes that are converted to iNeurons go through a proliferative stage. Given that Ascl1 can induce neural progenitor cells to proliferate in permissive environments in which Notch signaling is active (Castro et al., 2011; Li et al., 2014), we asked whether the overexpression of Ascl1 in adult astrocytes triggered re-entry into the cell cycle. Notably, we performed these studies only at 4 dpi given the recent demonstration that BrdU inhibits astrocyte to neuron conversion when administered for longer periods (Wang et al., 2022). Both Ascl1 (2.55 ± 0.38%) and Ascl1*^SA^*^6^ (4.93 ± 0.66%) induced 8.2-fold and 15.8-fold increases in BrdU incorporation in zsGreen^+^ transduced cells relative to the iCre control transduction (0.31 ± 0.18%), respectively (Figures 3H,I). Moreover, even after only 4 dpi, there were small but significant 3.4-fold and 3.9-fold increases in the ratio of zsGreen transduced cells expressing NeuN after transduction with Ascl1 (3.89 ± 0.09%) and Ascl1*^SA^*^6^ (4.47 ± 0.14%) compared to iCre controls (1.15 ± 0.10%), respectively (Figures 3H,J). However, the percentage of proliferating cells remains very low, less than 5%, in both instances, either because only a subset of cells are induced to proliferate, or because cells that incorporate BrdU undergo cell death (Wang et al., 2022).

In summary, the AAV-GFAP-iCre system that we employed can be used to express Ascl1 in cortical astrocytes, and to trigger Cre-dependent reporter expression, which in turn can be used to trace the fate of transduced cells in the adult cerebral cortex.

### Mutating serine phospho-acceptor sites in *Ascl1* augments neuronal lineage conversion in the adult cortex

**FIGURE 4:**
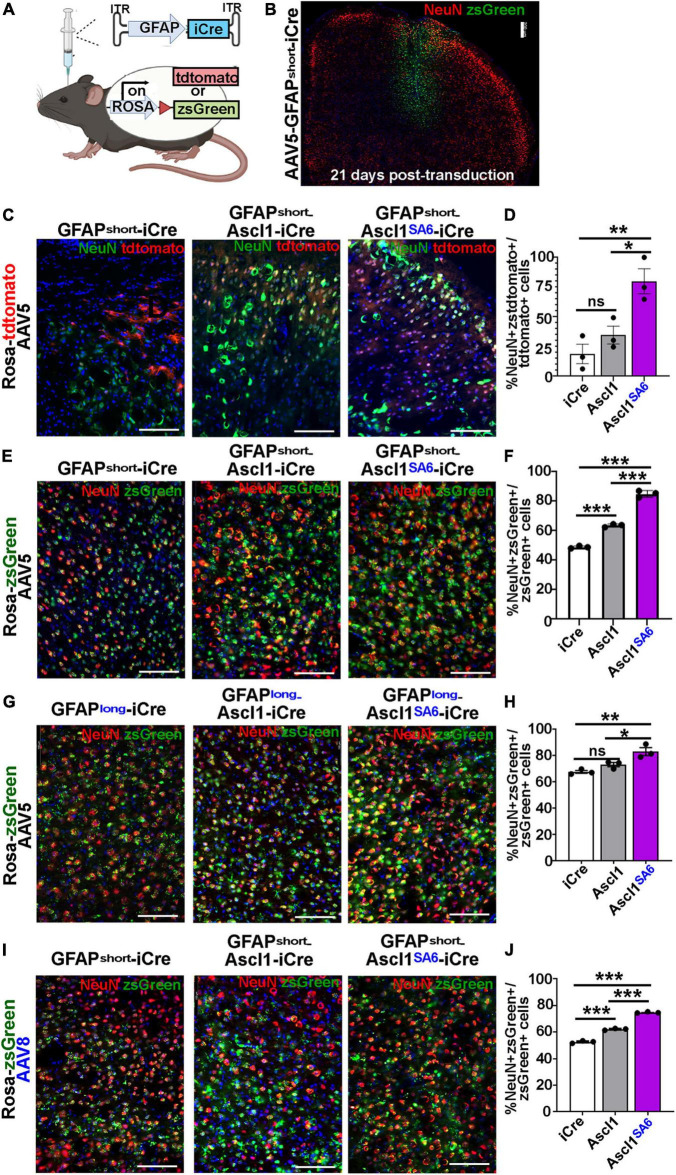
Ascl1*^SA^*^6^ induces more transduced cortical cells to express NeuN, a mature neuronal marker, than Ascl1. **(A)** Schematic illustration of Cre-based lineage tracing strategy, using AAV5-GFAPshort vectors and Rosa-tdtomato or Rosa-zsGreen transgenic animals. **(B)** Low magnification image of Rosa-zsGreen cortex transduced with AAV5-GFAPshort-iCre at 21 days post-transduction, showing zsGreen epifluorescence and NeuN expression. **(C)** Rosa-tdtomato cortex transduced with AAV5-GFAPshort-iCre, AAV5-GFAPshort-Ascl1-t2a-iCre, and AAV5-GFAPshort-Ascl1*^SA^*^6^-t2a-iCre at 21 days post-transduction, showing tdtomato epifluorescence and NeuN expression. **(D)** Quantification of the percentage of tdtomato^+^ cells that co-express NeuN. **(E)** Rosa-zsGreen cortex transduced with AAV5-GFAPshort-iCre, AAV5-GFAPshort-Ascl1-t2a-iCre, and AAV5-GFAPshort-Ascl1*^SA^*^6^-t2a-iCre at 21 days post-transduction, showing zsGreen epifluorescence and NeuN expression. **(F)** Quantification of the percentage of zsGreen^+^ cells that co-express NeuN. **(G)** Rosa-zsGreen cortex transduced with AAV5-GFAPlong-iCre, AAV5-GFAPlong-Ascl1-t2a-iCre, and AAV5-GFAPlong-Ascl1*^SA^*^6^-t2a-iCre at 21 days post-transduction, showing zsGreen epifluorescence and NeuN expression. **(H)** Quantification of the percentage of zsGreen^+^ cells that co-express NeuN. **(I)** Rosa-zsGreen cortex transduced with AAV8-GFAPshort-iCre, AAV8-GFAPshort-Ascl1-t2a-iCre, and AAV8-GFAPshort-Ascl1*^SA^*^6^-t2a-iCre at 21 days post-transduction, showing zsGreen epifluorescence and NeuN expression. **(J)** Quantification of the percentage of zsGreen^+^ cells that co-express NeuN. Scale bars in panel **B** = 200 μm, and panels **C,E,G,I** = 100 μm. Significance was defined as *p*-values less than 0.05 and denoted as follows: ns, not significant, <0.05 *, <0.01 **, <0.001 ***.

*In vivo* astrocyte-to-neuron lineage conversion has been reported using different AAVs (AAV5 or AAV8), which have both been reported to transduce cortical astrocytes (Aschauer et al., 2013), and using a 681bp human gfaABC(1)D promoter (Lee et al., 2008; Livingston et al., 2020) (hereafter, GFAPshort) or the 2.2 kb GFAPlong promoter described above (Barker et al., 2018; Chen et al., 2020; Livingston et al., 2020; Puls et al., 2020). We thus questioned which promoter and AAV delivery system was optimal. Notably, the GFAPshort promoter shows similar astrocyte-specificity as a 2.2 kb GFAPlong promoter, but drives two-fold higher levels of gene expression (Lee et al., 2008). We thus compared AAV5 and AAV8 capsids containing GFAPlong and GFAPshort promoters (Lee et al., 2008), and transduced Rosa-zsGreen and Rosa-tdtomato reporter mice, two of the brightest fluorescent reporters (Madisen et al., 2010) (Figure 4). Each comparative group had a set of three genetic cargos: iCre alone (control), Ascl1-iCre, or Ascl1*^SA^*^6^-iCre. Our three comparisons were AAV8 vs. AAV5 with the short GFAP promoter, GFAPshort vs. GFAPlong in AAV5, and Rosa-tdtomato vs. Rosa-zsGreen using AAV5-GFAP short constructs. As above, packaged AAVs (4.8 × 10^9^ GC total in a 1 uL total volume) were stereotactically injected into the motor cortex using the same coordinates (AP: + 2.15, L/M: ± 1.7, DV: −1.7).

We first compared the ability of AAV5-GFAPshort constructs to induce NeuN expression when injected into the cortex of Rosa-tdtomato mice (Figures 4C,D) and Rosa-zsGreen (Figures 4E,F) mice. In Rosa-tdtomato cortices analyzed at 21 dpi, statistically similar numbers of iCre (18.8 ± 8.1%) and Ascl1 (34.7 ± 7.5%) transduced tdtomato^+^ cells expressed NeuN, while Ascl1*^SA^*^6^ transduction induced a 4.3-fold increase in the number of NeuN expressing tdtomato^+^ cells (79.9 ± 10.5%) compared to iCre “baseline” levels (Figures 4C,D). We then compared the same AAV5-GFAPshort constructs transduced into motor cortices of Rosa-zsGreen mice. In Rosa-zsGreen cortices analyzed at 21 dpi, Ascl1 (63.6 ± 0.6%) and Ascl1*^SA^*^6^ (85.0 ± 1.3%) induced 1.3- and 1.7-fold increases, respectively, in the number of tdtomato^+^ cells expressing NeuN compared to iCre control levels (48.9 ± 0.5%) (Figures 4E,F). Thus, in both reporter mice, Ascl1*^SA^*^6^ was more efficient at inducing NeuN expression compared to Ascl1, but given that zsGreen expression appeared more widespread than tdtomato, we used Rosa-zsGreen mice for the remainder of the study.

We next assessed neuronal marker expression induced by AAV5-GFAPlong constructs introduced into Rosa-zsGreen motor cortices (Figures 4G,H). At 21 dpi, 67.7 ± 1.1% of the iCre control transduced zsGreen^+^ cells expressed NeuN (Figures 4G,H). Compared to AAV5-GFAPshort-iCre constructs, the AAV5-GFAPlong-iCre vector induced a 1.4-fold increase in “background” reporter expression in neurons, suggesting that the long promoter is less astrocyte-specific. Nevertheless, when Ascl1*^SA^*^6^ was expressed from the GFAPlong promoter, 1.2-fold more zsGreen^+^ cells expressed NeuN (83.2 ± 2.9%) compared to iCre, whereas Ascl1 neuronal conversion rates (73.2 ± 1.6%) were not above iCre baseline (67.7 ± 1.1%) (Figures 4G,H).

Next, we compared the AAV8 capsid using the GFAPshort promoter. With this system, we also found that 52.6 ± 0.6% of iCre control transduced zsGreen^+^ cells expressed NeuN, but both Ascl1 (62.2 ± 0.4%), and more strikingly, Ascl1*^SA^*^6^ (74.8 ± 0.3%) induced significant 1.2- and 1.4-fold increases, respectively in the number of zsGreen^+^ cells expressing NeuN at 21 dpi (Figures 4I,J). From these studies, we conclude that Ascl1*^SA^*^6^ transduced cells more frequently express NeuN compared to Ascl1 transduced cells when delivered to the adult motor cortex using GFAP promoter elements. In addition, our study supports previous studies using transgenic mice that suggested that the GFAPshort promoter is more specific to cortical astrocytes than the GFAPlong promoter (Lee et al., 2008). Finally, compared to AAV8, the AAV5 capsid labels fewer cortical neurons when GFAP-iCre sequences are included, and may thus be better suited for initial astrocyte targeting and neuronal reprogramming *in vivo.*

### Ascl1*^SA^*^6^ and to a lesser extent Ascl1 downregulates astrocytic marker expression

True lineage conversion requires that targeted cells, in our case astrocytes, not only turn on neuronal markers, but also extinguish the expression of glial markers. Indeed, in the embryonic cortex *in vivo* (Li et al., 2014) and in neuroblastoma (Woods et al., 2022) and glioblastoma (Azzarelli et al., 2022) cells *in vitro*, Ascl1*^SA^*^6^ is more efficient at turning on neuronal gene expression and less efficient at transactivating the Sox9 glial promoter compared to Ascl1 (Li et al., 2014). Here, we thus asked whether in the adult cortex, Ascl1*^SA^*^6^ could more efficiently downregulate Sox9 and GFAP expression in mature astrocytes. In this set of experiments, we compared the AAV5 vector carrying GFAPlong and GFAPshort promoters and the AAV8 vector with the GFAPshort promoter. As above, packaged AAVs (4.8 × 10^9^ GC total in a 1 uL total volume) were stereotactically injected into the motor cortex of Rosa-zsGreen animals using the same coordinates (AP: + 2.15, L/M: ± 1.7, DV: −1.7), and brains were harvested at 21 dpi.

**FIGURE 5:**
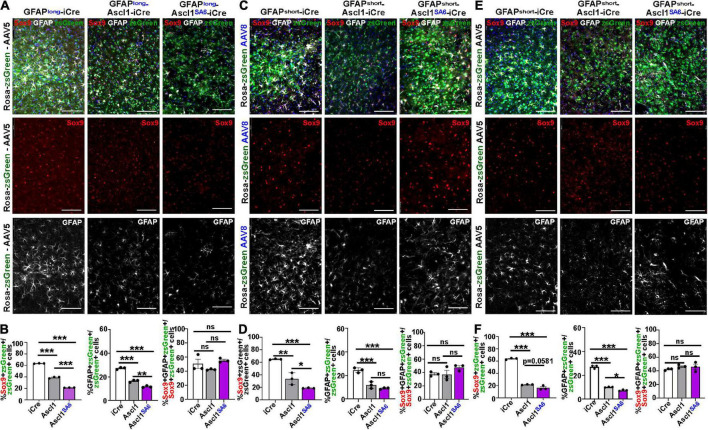
Ascl1*^SA^*^6^ more efficiently represses astrocytic markers Sox9 and GFAP in transduced cortical cells compared to Ascl1. **(A)** Rosa-zsGreen cortex transduced with AAV5-GFAPlong-iCre, AAV5-GFAPlong-Ascl1-t2a-iCre, and AAV5-GFAPlong-Ascl1*^SA^*^6^-t2a-iCre at 21 days post-transduction, showing zsGreen epifluorescence, Sox9 (red) and GFAP (white) expression in merged and separate channels. Blue is a DAPI counterstain. **(B)** Quantification of the percentage of zsGreen^+^ cells that co-express Sox9 or GFAP, and the percentage of zsGreen^+^Sox9^+^ cells that co-express GFAP. **(C)** Rosa-zsGreen cortex transduced with AAV5-GFAPshort-iCre, AAV5-GFAPshort-Ascl1-t2a-iCre, and AAV5-GFAPshort-Ascl1*^SA^*^6^-t2a-iCre at 21 days post-transduction, showing zsGreen epifluorescence, Sox9 (red) and GFAP (white) expression in merged and separate channels. Blue is a DAPI counterstain. **(D)** Quantification of the percentage of zsGreen^+^ cells that co-express Sox9 or GFAP, and the percentage of zsGreen^+^Sox9^+^ cells that co-express GFAP. **(E)** Rosa-zsGreen cortex transduced with AAV8-GFAPshort-iCre, AAV8-GFAPshort-Ascl1-t2a-iCre, and AAV8-GFAPshort-Ascl1*^SA^*^6^-t2a-iCre at 21 days post-transduction, showing zsGreen epifluorescence, Sox9 (red) and GFAP (white) expression in merged and separate channels. Blue is a DAPI counterstain. **(F)** Quantification of the percentage of zsGreen^+^ cells that co-express Sox9 or GFAP, and the percentage of zsGreen^+^Sox9^+^ cells that co-express GFAP. Scale bars = 100 μm. Significance was defined as *p*-values less than 0.05 and denoted as follows: ns, not significant, <0.05 *, <0.01 **, <0.001 ***.

As expected, the majority of iCre-transduced cells expressed Sox9, an astrocytic marker, after 21 dpi regardless of whether iCre was expressed with AAV5-GFAP-long (63.7 ± 0.2%) (Figures 5A,B), AAV8-GFAPshort (65.1 ± 0.5%) (Figures 5C,D), or AAV5-GFAPshort (64.0 ± 0.8%) (Figures 5E,F) vectors, confirming astrocytic targeting of a large proportion of cells. However, the ratio of iCre control cells that co-expressed GFAP was lower than Sox9 for all vectors, including AAV5-GFAP-long (27.3 ± 0.5%) (Figures 5A,B), AAV8-GFAPshort (24.5 ± 1.2%) (Figures 5C,D) or AAV5-GFAPshort (27.2 ± 0.8%) (Figures 5E,F). One possibility is that astrocytes that initially expressed GFAP at the time of transduction turned off their GFAP expression within the 21 days before analysis, or alternatively, GFAP may be transcribed and not translated. Nevertheless, regardless of this discrepancy, based on Sox9 expression, we can conclude that over half of iCre control-transduced cells are astrocytes at 21 dpi.

We next assessed Sox9/zsGreen co-expression 21 days after transduction of Ascl1, revealing 1.62-, 1.92- and 2.94-fold reductions, respectively, using AAV5-GFAP-long (39.3 ± 0.8%) (Figures 5A,B), AAV8-GFAPshort (34.0 ± 5.5%) (Figures 5C,D) or AAV5-GFAPshort (21.8 ± 0.3%) (Figures 5E,F) vectors. In all cases, Ascl1*^SA^*^6^ reduced Sox9/zsGreen co-expression levels even further, with 2.95-, 3.43- and 4.65-fold reductions, respectively using AAV5-GFAP-long (21.6 ± 0.1%) (Figures 5A,B), AAV8-GFAPshort (19.0 ± 0.3%) (Figures 5C,D) or AAV5-GFAPshort (16.5 ± 2.0%) (Figures 5E,F) vectors.

Similarly, an analysis of GFAP/zsGreen co-expression at 21 dpi revealed 1.65-, 2.08- and 2.68-fold reductions, respectively, using AAV5-GFAP-long (16.6 ± 0.6%) (Figures 5A,B), AAV8-GFAPshort (11.8 ± 1.6%) (Figures 5C,D) or AAV5-GFAPshort (10.1 ± 0.2%) (Figures 5E,F) vectors. More pronounced 2.32- and 3.66-fold reductions in GFAP/zsGreen co-expression were observed at 21 dpi using AAV5-GFAP-long (11.8 ± 0.4%) (Figures 5A,B) and AAV5-GFAP short (7.4 ± 0.3%) (Figures 5E,F) vectors, respectively, to express Ascl1*^SA^*^6^. However, overexpression of Ascl1*^SA^*^6^ using AAV8-GFAPshort gave a similar 2.72-fold reduction in GFAP/zsGreen co-expression (9.0 ± 0.4%) as seen with Ascl1 (Figures 5C,D). Notably, the reduction in astrocytic marker was not due to changes in the ratio of Sox9^+^ cells that co-expressed GFAP, so both Sox9 single^+^ and Sox9/GFAP double^+^ cells were equally affected (Figures 5B,D,F). Taken together, these data support the contention that Ascl1 and Ascl1*^SA^*^6^ both suppress an astrocytic fate in the adult cortex, but Ascl1*^SA^*^6^ is more efficient at glial repression, similar to studies in the embryonic cortex (Li et al., 2014).

### Few Ascl1 and Ascl1*^SA^*^6^ transduced cells go through a Dcx^+^ neuroblast stage

It has been suggested that neuronal lineage conversion *in vivo* should include a transitory, immature Dcx^+^ neuroblast stage, as has been shown *in vitro* (Bocchi et al., 2021). We thus examined Dcx expression following the overexpression of Ascl1 and Ascl1*^SA^*^6^ in motor cortex astrocytes, again comparing the AAV5 vector carrying GFAPlong and GFAPshort promoters and the AAV8 vector with the GFAPshort promoter using the same coordinates and dosage, and brains were harvested at 21 dpi.

**FIGURE 6:**
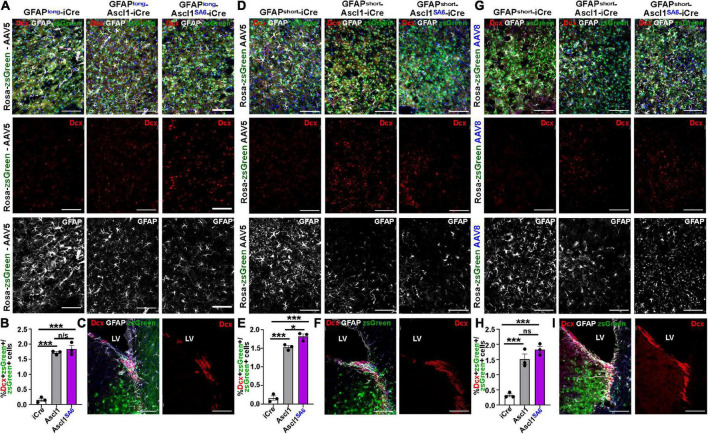
Limited induction of Dcx expression in GFAP^+^ transduced astrocytes by Ascl1 and Ascl1*^SA^*^6^. **(A–C)** Rosa-zsGreen cortex transduced with AAV5-GFAPlong-iCre, AAV5-GFAPlong-Ascl1-t2a-iCre, and AAV5-GFAPlong-Ascl1*^SA^*^6^-t2a-iCre at 21 days post-transduction, showing zsGreen epifluorescence, Dcx (red) and GFAP (white) expression in the parenchyma of the motor cortex **(A)** or in the ventricular-subventricular zone (V-SVZ) **(C)**. Blue is DAPI counterstain. **(B)** Quantification of the percentage of zsGreen^+^ cells that co-express Dcx. **(D–F)** Rosa-zsGreen cortex transduced with AAV5-GFAPshort-iCre, AAV5-GFAPshort-Ascl1-t2a-iCre, and AAV5-GFAPshort-Ascl1*^SA^*^6^-t2a-iCre at 21 days post-transduction, showing zsGreen epifluorescence, Dcx (red) and GFAP (white) expression in the parenchyma of the motor cortex **(D)** or in the ventricular-subventricular zone (V-SVZ) **(F)**. Blue is DAPI counterstain. Quantification of the percentage of zsGreen^+^ cells that co-express Dcx **(E)**. **(G–I)** Rosa-zsGreen cortex transduced with AAV8-GFAPshort-iCre, AAV8-GFAPshort-Ascl1-t2a-iCre, and AAV8-GFAPshort-Ascl1*^SA^*^6^-t2a-iCre at 21 days post-transduction, showing zsGreen epifluorescence, Dcx (red) and GFAP (white) expression in the parenchyma of the motor cortex **(G)** or in the ventricular-subventricular zone (V-SVZ) **(I)**. Blue is DAPI counterstain. Quantification of the percentage of zsGreen^+^ cells that co-express Dcx **(H)**. Scale bars = 100 μm. LV, lateral ventricle. Significance was defined as *p*-values less than 0.05 and denoted as follows: ns, not significant, <0.05 *, <0.01 **, <0.001 ***.

As expected, very few iCre-transduced cells expressed Dcx after 21 dpi regardless of whether iCre was expressed with AAV5-GFAP-long (0.16 ± 0.05%) (Figures 6A,B), AAV8-GFAPshort (0.33 ± 0.05%) (Figures 6G,H), or AAV5-GFAPshort (0.17 ± 0.06%) (Figures 6D,E) vectors. After 21 dpi, there were 10.9-, 4.72-, and 9.20-fold increases in Dcx/zsGreen co-expression following the overexpression of Ascl1 using AAV5-GFAP-long (1.72 ± 0.04%) (Figures 6A,B), AAV8-GFAPshort (1.52 ± 0.17%) (Figures 6G,H) or AAV5-GFAPshort (1.53 ± 0.04%) (Figures 6D,E) vectors, respectively, reflecting a very small fraction of the total transduced cells. Similarly, Ascl1*^SA^*^6^ induced 11.67-, 5.64-, and 10.94-fold increases in Dcx/zsGreen co-expression when delivered to the motor cortex using AAV5-GFAP-long (1.84 ± 0.12%) (Figures 6A,B), AAV8-GFAPshort (1.83 ± 0.11%) (Figures 6G,H) or AAV5-GFAPshort (1.83 ± 0.06%) (Figures 6D,E) vectors, respectively. However, with the exception of AAV5-GFAPshort, Ascl1*^SA^*^6^ was not better than Ascl1 at inducing Dcx expression. Notably, we confirmed that Dcx antibody staining was correct, as strong expression was seen in the V-SVZ, where neuroblasts are generated (Figures 6C,F,I).

Taken together, these data suggest that most transduced cells do not undergo a Dcx neuroblast stage, or that this stage is very transitory.

### Ascl1*^SA^*^6^ induces electrophysiological properties of iNeurons in targeted astrocytes

**FIGURE 7:**
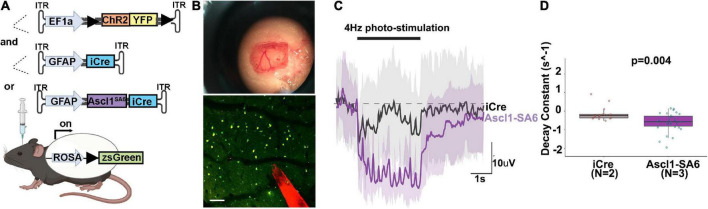
Optogenetic stimulation of ChR2 actuator reveals that Ascl1*^SA^*^6^ transduced cells have a shorter decay constant. **(A)** Schematic illustration of optogenetic experiment in which we injected AAVs carrying ChR2-(H134R)-YFP, an optogenetic actuator, and co-transduced AAV5-GFAPshort-iCre or AAV5-GFAPshort-Ascl1*^SA^*^6^-iCre into the cortex of Rosa-zsGreen mice. A 4 weeks later, photostimulation experiments were performed. **(B)** Craniotomy and cortical window used for simultaneous photostimulation and electrophysiological recordings. Also shown is a two-photon z-stack projection of the cortical tissue showing zsGreen^+^ cells (green channel) and the tungsten electrode tip (red channel); the circular yellow area represents the site of stimulation near the electrode; scalebar = 100 μm. **(C)** Representative raw voltage traces showing the changes in the LFP band following 3 s of photostimulation at 4 Hz for both Ascl1*^SA^*^6^ (purple trace) and iCre (black trace) treatments. Shaded areas indicate the voltage standard deviation measured across different repetitions, within the same photostimulated area. **(D)** Measurement of the decay constant of photostimulated cells in iCre and Ascl1*^SA^*^6^ transduced brains. Dots in the boxplots indicate individual measures and “N” indicates the number of mice used in each group. Significance was defined as *p*-values less than 0.05 and denoted as follows: ns, not significant, <0.05 *, <0.01 **, <0.001 ***.

To promote functional recovery in pathological conditions, iNeurons must integrate into existing neural circuits by making synaptic connections with endogenous neurons and sending axons to appropriate neuronal targets. To test neural network integration of iNeurons *in vivo*, we co-transduced AAVs carrying GFAP-iCre or GFAP-Ascl1*^SA^*^6^ with FLEX-ChR2-(H134R)-YFP, a Cre-dependent optogenetic actuator that offers a sensitive way to photoactivate neurons and elicit large evoked potentials (Figure 7A). After 36 days, we made a cranial window and performed intracortical electrophysiological recordings to assess local field potentials, a measure of aggregate neuronal activity, in response to ChR2 photoactivation (20 Hz, 10 ms pulse length, 5s total) (Figure 7B). In a representative trace, and quantified for several sites, Ascl1*^SA^*^6^ iNeurons transduced cortices exhibited a faster decay of evoked potentials to baseline than did iCre transduced cortices (Figures 7C,D), a neuronal feature. This data thus supports the contention that Ascl1*^SA^*^6^ successfully converts transduced astrocytes into functional iNeurons.

## Discussion

In this study, we performed a detailed comparison of the capacity of Ascl1 and Ascl1*^SA^*^6^ to induce neuronal markers and suppress glial markers when expressed in adult cortical astrocytes *in vivo.* We found that with each combination of AAV capsids, GFAP promoters and Rosa-reporter lines tested, a higher proportion of Ascl1*^SA^*^6^ transduced cells consistently expressed NeuN, a mature neuronal marker, and acquired complex dendritic arbors compared to cells transduced with Ascl1 or iCre controls. In contrast, an equivalent, low number of Ascl1 and Ascl1*^SA^*^6^ transduced cells had the signature of a transitory Dcx^+^ neuroblast stage, suggesting that either this stage is very transitory, or not induced by these TFs. In addition, both Ascl1 and Ascl1*^SA^*^6^ could suppress the expression of astrocytic markers (Sox9 and GFAP), although Ascl1*^SA^*^6^ was again superior in this regard. The enhanced neurogenic capacity of Ascl1*^SA^*^6^ vs. Ascl1 is in keeping with prior studies in embryonic cortical progenitor cells *in vivo* (Li et al., 2014). In addition, it was recently demonstrated that ASCL1*^SA^*^5^ (note that the human ASCL1 gene has 5 SP sites) can induce a glioblastoma stem cell line to undergo terminal differentiation and exit the cell cycle more effectively than native ASCL1, leading to growth suppression of this tumor cell line (Azzarelli et al., 2022). Taken together with our work in the current study, there is now cumulative support for the enhanced pro-neurogenic and anti-astrocytic capacity of Ascl1*^SA^*^6^ vs. Ascl1.

Even though our data shows clear differences between Ascl1 and Ascl1*^SA^*^6^ in regulating neuronal marker expression when expressed in the adult brain, with an abundance of caution, it is important to acknowledge a recent debate created by several high-profile 2021 and 2022 publications that questioned whether brain glia (astrocytes, microglia) can be converted to neurons *in vivo* (Rao et al., 2021; Wang et al., 2021). Notably, with each of our strategies, incorporating different AAV capsids and GFAP promoters, our intent was to preferentially target cortical astrocytes without any leaky expression in endogenous neurons. However, similar to others, we observed a significant level of reporter expression in endogenous neurons, using either mCherry or iCre control vectors. Thus, we were not able to achieve the astrocytic specificity that we desired. Moving forward, it is important to address these concerns by incorporating robust lineage tracing of the starter glial population, and by pre-labeling endogenous neurons, as outlined in a new position paper (Bocchi et al., 2021).

Notably, Wang et al. (2021) found that the TF coding sequences act in cis to alter the astrocyte specificity of the GFAP promoter, an experimental confound that is enhanced over time, as astrocytic-specificity is initially observed at 4 days post-transduction, even with a GFAP-Neurod1-mCherry vector (Wang et al., 2021). Presumably, the same cis-effects of the Ascl1 cargo are taking place in our system. In this regard, it is interesting that bHLH TFs suppress the GFAP promoter indirectly by sopping up glial cofactors, such as CBP-SMAD, and preventing STAT activation, all of which are required to transactivate the GFAP promoter (Sun et al., 2001). One possibility is that this indirect mode of suppression of glial gene expression may account for some of the reduced Sox9 and GFAP expression induced by Ascl1 and Ascl1*^SA^*^6^. However, as Ascl1 and Ascl1*^SA^*^6^ differ in only six codons, it seems unlikely that the enhanced capacity of Ascl1*^SA^*^6^ to turn on neuronal genes and turn off glial genes is solely due to the cis-activity of these two genes being significantly different. For instance, in addition to sopping up glial cofactors, Ascl1 and Ascl1*^SA^*^6^ may suppress glial genes by inducing the expression of downstream transcriptional repressors, an indirect mode of action that was previously attributed to Neurog2 during cortical development (Kovach et al., 2013). Regardless of how Ascl1 and Ascl1*^SA^*^6^ function during neuronal reprogramming, astrocytic suppression by the proneural bHLH TFs Neurog2 and Ascl1 has been firmly established in the embryonic CNS (Oproescu et al., 2021). Indeed, embryonic cortical progenitors have a reduced propensity to differentiate into astrocytes, based on *in vitro* differentiation assays or *in vivo* lineage tracing (Han et al., 2021). We thus favor the model that Ascl1*^SA^*^6^ can both suppress astrocytic gene expression and transactivate neuronal genes more efficiently than Ascl1, as we showed definitively in the embryonic brain (Li et al., 2014).

The Rao et al. (2021) study highlights a different concern, as their manuscript contradicted an earlier claim that microglia could be converted to iNeurons *in vivo* (Matsuda et al., 2019). In a published response by the authors of the initial microglia-to-iNeuron conversion paper, the authors suggested that the lentiviral delivery strategy used by Rao et al. (2021) achieved Neurod1 expression at a magnitude lower than what is required for successful neuronal reprogramming (Matsuda and Nakashima, 2021). However, a recent report using glial lineage tracing similarly suggested that Neurod1 has a limited capacity to convert brain astrocytes to iNeurons (Leib et al., 2022). Nevertheless, the importance of achieving threshold levels of the bHLH TFs has similarly been shown in the embryonic brain, with Neurog2 not able to convert ventral telencephalic progenitors to a dorsal cortical fate in Ascl1*^Neurog^*^2*KI*^ mice (Parras et al., 2002), whereas high levels of Neurog2 expression achieved by *in utero* electroporation of the ventral telencephalon effectively induces a cortical fate in ventral domains (Kovach et al., 2013). Thus, levels of bHLH TF expression are indeed important to how these genes function and their capacity to turn on downstream genes.

As a final comment, even though the GFAP promoter may drive background labeling of endogenous neurons, it does not negate the capacity of glia to be converted to neurons, as shown definitively using retroviruses *in vivo*, and supported by hundreds of *in vitro* studies (Barker et al., 2018; Sharif et al., 2021; Vasan et al., 2021). In our study in the adult brain, it is possible that the astrocytes we targeted are resident cells in the brain parenchyma, or newly generated reactive astrocytes derived from V-SVZ neural stem cells, as shown recently (Faiz et al., 2015). Lineage tracing of V-SVZ cells using nestin-Cre*^ERT^*^2^ (Faiz et al., 2015) and of resident astrocytes using Aldh1l1-Cre*^ERT^*^2^ (Srinivasan et al., 2016) could help to distinguish the source of new neurons. Indeed, in December 2021, a position paper listed important new obligatory controls for *in vivo* neuronal reprogramming, designed to address recent controversies in the field, including: lineage tracing (neuronal and glial), lineage trajectory analyses (single cell transcriptomic studies) and functional assessments of iNeuron activity (Bocchi et al., 2021).

As a final statement, in support of the therapeutic power of neuronal reprogramming, new studies demonstrating that the beneficial effects of neuronal reprogramming are lost upon chemogenetic silencing or ablation of new neurons in Parkinson’s disease (Qian et al., 2020) and stroke (Irie et al., 2021) models, respectively, provide growing support for the potential therapeutic power of endogenous neuronal replacement.

## Data availability statement

The raw data supporting the conclusions of this article will be made available by the authors, without undue reservation.

## Ethics statement

The animal study was reviewed and approved by the Sunnybrook Research Institute Comparative Research Animal Care Committee (ACC).

## Author contributions

HG and EP did the conceptualization, carried out the data curation and formal analysis, investigated, visualized, and validated the data, performed the methodology, wrote the original draft, and wrote, reviewed, and edited the manuscript. DZ carried out the formal analysis, investigated and validated the data, performed the methodology, and wrote, reviewed, and edited the manuscript. JaM, LV, and AT carried out the data curation and formal analysis, investigated and validated the data, performed the methodology, and wrote, reviewed, and edited the manuscript. FS, TF, and VC carried out the data curation, performed the methodology, validated the data, and wrote, reviewed, and edited the manuscript. ML investigated and validated the data, performed the methodology, and wrote, reviewed, and edited the manuscript. OP investigated the data, performed the methodology, and wrote, reviewed, and edited the manuscript. NK carried out the formal analysis and investigated the data. DK, MF, BS, and JM carried out the resources, supervised the data, and wrote, reviewed, and edited the manuscript. CS carried out the funding acquisition, project administration, and resources, did the conceptualization, supervised and validated the data, wrote the original draft, and wrote, reviewed, and edited the manuscript. All authors contributed to the article and approved the submitted version.
