# Supplementary material for: Ascl1 phospho-site mutations enhance neuronal conversion of adult cortical astrocytes in vivo
Source: Front Neurosci. 2022 Aug 18;16:917071. doi: 10.3389/fnins.2022.917071 (PMC9434350; doi:10.3389/fnins.2022.917071)
Supplement: Supplementary file 1 [file Image_1.pdf]

Supplemental Figures

Figure S1. Related to Figure 1. (A)Western blot analysis of lysates from left (sample 1) and right (sample 2) cortical hemispheres transduced with AAV8-GFAPlong-mCherry (mCh), AAV8-GFAPlong-Ascl1-mCherry orAAV8-GFAPlong-Ascl1^{SA6}-mCherry and analyzed for the expression of phospho-Ascl1, Ascl1 (BD Bio) and ß-actin in mCherry, Ascl1 and Ascl1^{SA6}-transduced brains at 7 days post infection (dpi). (B) Densitometric quantification of expression levels of Ascl1 (BD Bio) normalized to ß-actin, and relative phospho-Ascl1 levels normalized to total Ascl1 and ß-actin. (C) Western blot analysis of E14.5 dorsal (dTel) and ventral (vTel) telencephalic lysates blotted with the BD Bio Ascl1 antibody. (D) Western blot analysis of phospho-Ascl1, Ascl1 (Abcam) and ß-actin in lysates from left (sample 1) and right (sample 2) cortical hemispheres transduced with mCherry, Ascl1 and Ascl1^{SA6}-transduced brains at 7 dpi.

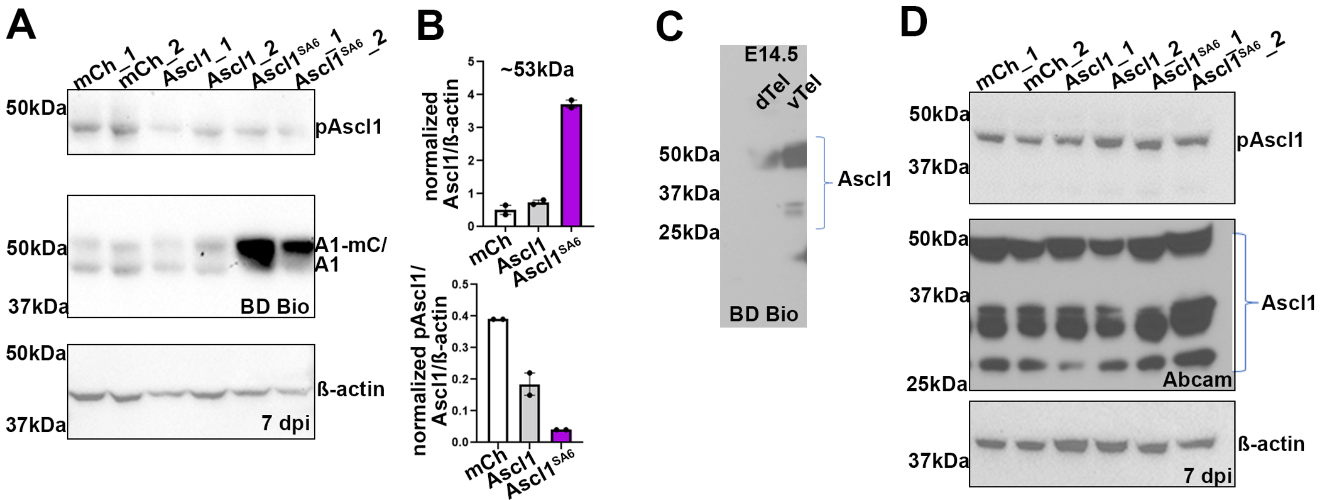

A
mCh_1
mCh_2
Ascl1_1
Ascl1_2
Ascl1SA6_1
Ascl1SA6_2
50kDa
pAscl1
50kDa
A1-mC/
A1
37kDa
BD Bio
50kDa
ß-actin
37kDa
7 dpi
B
~53kDa
normalized
Ascl1/ß-actin
mCh
Ascl1
Ascl1SA6
normalized pAscl1/
Ascl1/ß-actin
C
E14.5
dTel
vTel
50kDa
37kDa
25kDa
Ascl1
BD Bio
D
pAscl1
37kDa
Abcam
25kDa
7 dpi
